# ﻿Two new species of *Cerviniella* Smirnov, 1946 (Copepoda, Harpacticoida, Aegisthidae) from the Yellow Sea, Korea

**DOI:** 10.3897/zookeys.1178.105407

**Published:** 2023-09-07

**Authors:** Kyuhee Cho, Jong Guk Kim, Jimin Lee

**Affiliations:** 1 Ocean Climate Response & Ecosystem Research Department, Korea Institute of Ocean Science & Technology, Busan 49111, Republic of Korea Korea Institute of Ocean Science & Technology Busan Republic of Korea; 2 Division of Zoology, Honam National Institute of Biological Resources, Mokpo 58762, Republic of Korea Honam National Institute of Biological Resources Mokpo Republic of Korea

**Keywords:** Cerviniellinae, Crustacea, Korean fauna, meiofauna, Yellow Sea

## Abstract

Two new species, *Cerviniellabisegmenta***sp. nov.** and *C.permixta***sp. nov.**, are described in detail with illustrations based on females from the Korean Yellow Sea. These species lacking the fourth leg endopod belong to the *mirabilipes* group, one of two species groups within the genus *Cerviniella* Smirnov, 1946. Both species can be distinguished from each other by the surface ornamentation of the cephalothorax, shape of the rostrum tip, antennule segments, armature formula of thoracic legs 1–4, and length ratio of the caudal rami. *Cerviniellabisegmenta***sp. nov.** is characterized by a short caudal ramus and a two-segmented antennary exopod, which are unique within the genus. *Cerviniellapermixta***sp. nov.** differs from other congeners of the *mirabilipes* group by the seven-segmented antennule, the armature formulae of the exopod of the antenna and thoracic legs 1–4, and the modified apical inner element of the second endopodal segment of the second leg. The present study is the first to identify the genus *Cerviniella* in Korean waters, resulting in extension of its distribution area to East Asia.

## ﻿Introduction

The genus *Cerviniella* Smirnov, 1946 was established by Smirnov (1946) for *Cerviniellamirabilipes* Smirnov, 1946, and originally placed in the family Cerviniidae Sars, 1903. However, subsequent phylogenetic studies have changed its taxonomic position. Seifried and Schminke (2003) reclassified Cerviniidae as a subfamily with the family Aegisthidae Giesbrecht, 1893, which includes two other subfamilies, Aegisthinae Giesbrecht, 1893, and Cerviniopseinae Brodskaya, 1963. For the past two decades, the genus *Cerviniella* has been considered a member of the subfamily Cerviniinae Sars, 1903. However, based on a recent genus-level molecular phylogenetic and morphological study of Aegisthidae, Khodami et al. (2020) suggested that *Cerviniella* should be included in the subfamily Cerviniellinae Khodami, Mercado-Salas & Martínez Arbizu, 2020. The investigators suggested that the three inbenthic genera, *Cerviniella*, *Eucanuella* Scott T., 1901, and *Hase* Corgosinho, Kihara, Schizas, Ostmann, Martínez Arbizu & Ivanenko, 2018, are distinct from the other three epibenthic cervinnid genera, *Cervinia* Norman in Brady, 1878, *Expansicervinia* Montagna, 1981, and *Paracerviniella* Brodsky, 1963. The genus *Cerviniella* was designated as the type genus of the new subfamily. The family Aegisthidae comprises 95 species, excluding species of the genera *Ameliotes* Por, 1969 and *Arcticocarella* Kornev & Chertoprud, 2008, within five subfamilies: Aegisthinae, Cerviniellinae, Cerviniinae, Cerviniopseinae, and Pontostratiotinae Scott A., 1909 (Khodami et al. 2020; Walter and Boxshall 2023).

In the 20^th^ and 21^st^ centuries, eight and five additional species of *Cerviniella* were described, respectively; therefore, 13 species have been identified thus far (Por 1964, 1969; Bodin 1968; Coull 1973; Becker 1974; Apostolov 2011; Kihara and Martínez Arbizu 2012). *Cerviniella* has adapted to a burrowing lifestyle; they have a robust body, short antennules, and inward-bent thoracic legs with strong outer spines. They are distributed in various regions, including the Arctic and Mediterranean regions around Europe, Atlantic regions around Europe and America, the Western Indian Ocean, and the Southwestern Pacific. They have a diverse bathymetric distribution from the coastal region to the deep sea (Apostolov 2011; Kihara and Martínez Arbizu 2012: table 1).

Apostolov (2011) subdivided the genus *Cerviniella* into the *brodskayae* and *mirabilipes* species groups, based on the presence or absence, respectively, of the P4 endopod. The *brodskayae* group consists of eight species: *C.brodskayae* Por, 1969, *C.bodini* Coull, 1973, *C.hamata* Coull, 1973, *C.peruana* Becker, 1974, *C.abyssalis* Apostolov, 2011, *C.longifurcata* Apostolov, 2011, *C.arctica* Kihara & Martínez Arbizu, 2012, and *C.hitoshii* Kihara & Martínez Arbizu, 2012. The *mirabilipes* group consists of five species: *C.mirabilipes* (type species), *C.talpa* (Por, 1964), *C.lagarderei* Bodin, 1968, *C.langi* Bodin, 1968, and *C.danae* Kihara & Martínez Arbizu, 2012. However, Kihara and Martínez Arbizu (2012) provided the amended generic diagnosis, which was unaware of Apostolov’s (2011) subdivision.

During a biodiversity study of benthic harpacticoids in Korea, we found two new species of the genus *Cerviniella* in the sublittoral zone of the Yellow Sea. Here, we discuss the morphological characteristics of the newly found species and provide a key to species of the genus *Cerviniella*.

## ﻿Materials and methods

Sediments were collected from the Yellow Sea using a Smith-McIntyre grab (0.1 m^2^) aboard R/V Eardo and R/V Onnuri of the Korea Institute of Ocean Science & Technology (**KIOST**). The upper sediment surface (0–5 cm) was subsampled and treated with 7.5% magnesium chloride (MgCl_2_) to anesthetize the benthic organisms. After 30 min, the samples were preserved in a 10% formalin/seawater solution. The Ludox centrifugation method was used to separate benthic organisms from sediments (Burgess 2001). Harpacticoids were separated from other benthic organisms under a stereomicroscope (M165 C; Leica Microsystems, Wetzlar, Germany) and preserved in 5% formalin. The *Cerviniella* specimens were dissected in lactic acid with tungsten needles, mounted on H-S slides (Double slide plate, BSDS-011R; Biosolution, Daegu, Republic of Korea) in lactophenol:glycerine (1:5) (cf. Shirayama et al. 1993), and sealed with transparent nail varnish. All drawings were made under a BX53 differential interference contrast light microscope (BX53; Olympus, Tokyo, Japan) with a camera lucida. Scale bars in the figures are indicated in micrometers.

The descriptive terminology was followed by Huys et al. (1996). The following abbreviations were used in the text and figure legends: **ae**, aesthetasc; **exp**, exopod; **enp**, endopod; P1–P6, first to sixth thoracopods; exp (enp)-1 (-2, -3), proximal (middle, distal) segment of a ramus. The type materials of two *Cerviniella* species were deposited in the Marine Biodiversity Institute of Korea (**MABIK**), Seocheon, Republic of Korea. Some materials were stored at the Marine Interstitial fauna Resources Bank (**MInRB**) of KIOST, Busan, Republic of Korea.

## ﻿Results

**Order Harpacticoida Sars, 1903**

**Family Aegisthidae Giesbrecht, 1893**

**Subfamily Cerviniellinae Khodami, Mercado-Salas & Martínez Arbizu, 2020**

### ﻿Genus Cerviniella

Taxon classificationAnimaliaHarpacticoidaAegisthidae

Smirnov, 1946

3D15F080-B211-593E-908C-33C59530C88E

#### Type species.

*Cerviniellamirabilipes* Smirnov, 1946.

#### Other species.

*Cerviniellaabyssalis* Apostolov, 2011, *C.arctica* Kihara & Martínez Arbizu, 2012, *C.bodini* Coull, 1973, *C.brodskayae* Por, 1969, *C.danae* Kihara & Martínez Arbizu, 2012, *C.hamata* Coull, 1973, *C.hitoshii* Kihara & Martínez Arbizu, 2012, *C.lagarderei* Bodin, 1968, *C.langi* Bodin, 1968, *C.longifurcata* Apostolov, 2011, *C.peruana* Becker, 1974, and *C.talpa* (Por, 1964).

### ﻿ Cerviniella bisegmenta sp. nov.

Taxon classificationAnimaliaHarpacticoidaAegisthidae

E7D46341-203F-58EF-B13D-055A37536DC4

https://zoobank.org/78650D35-782E-409D-9FE6-4FA038DF9680

Figs 1 , 2 , 3 , 4

#### Type locality.

The Yellow Sea; 34°59'40.14"N, 125°00'2.82"E; 88 m depth.

#### Type material.

***Holotype*.** One ♀ preserved in a vial with 80% ethanol (MABIK CR00253873); collected from the type locality, 20 April 2019. ***Paratypes*.** One ♀ (MABIK CR00253868) dissected on 11 slides, three ♀♀ (MABIK CR00253869–00253871) each dissected on 10 slides, six ♀♀ (MABIK CR00253874) and eight ♀♀ (MInRB-Hr88-L001) preserved in a vial with 80% ethanol, collection data as in holotype; S.L. Kim leg.

#### Description.

**Female** (based on the holotype and paratypes). ***Body*** length from anterior margin of rostrum to posterior margin of caudal rami (paratype, MABIK CR00253868, in lateral view, telescoping of somites not considered) 797 μm (range: 694–797 μm, *n* = 11, holotype: 765 μm).

**Figure 1.**
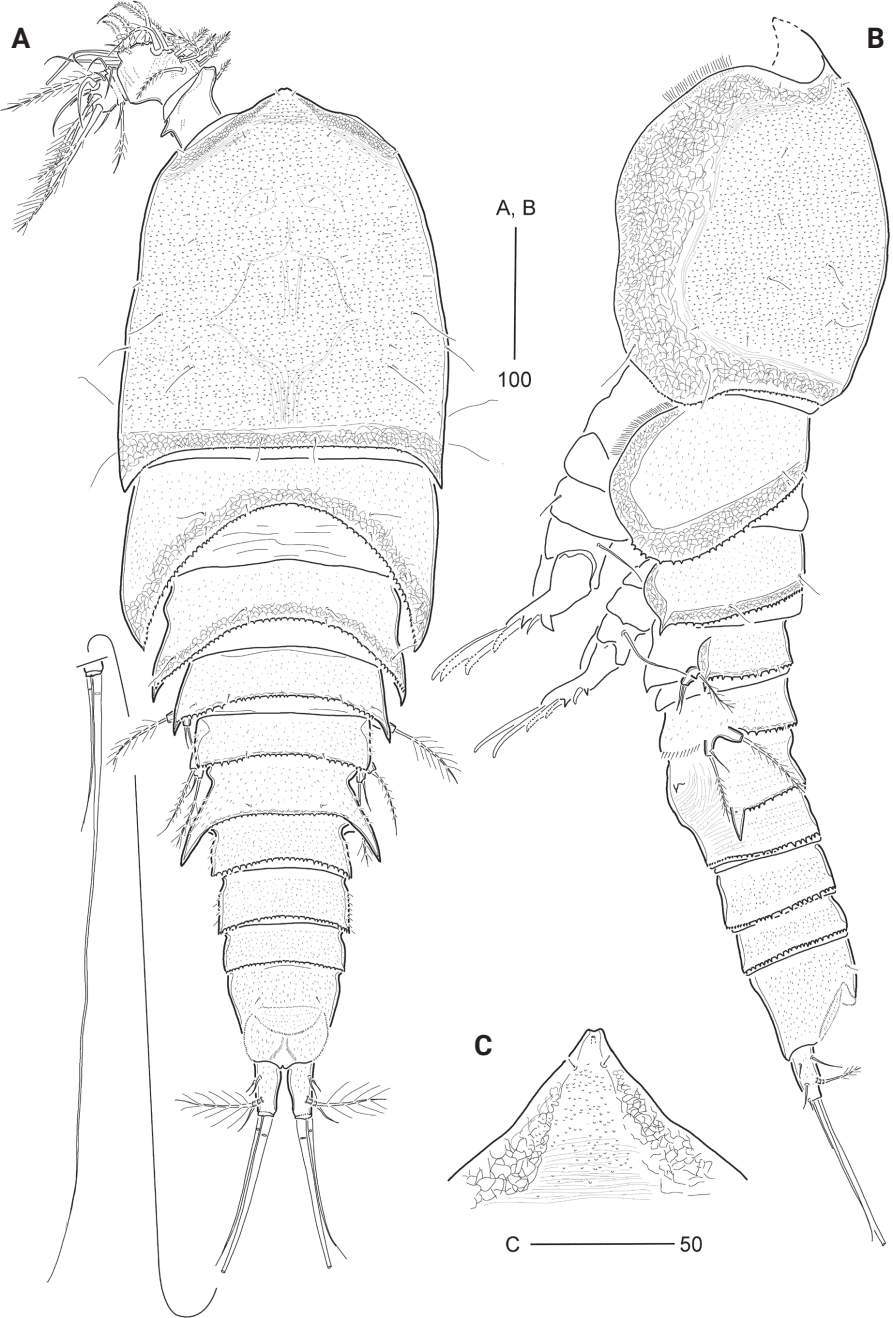
*Cerviniellabisegmenta* sp. nov. Female. Holotype (MABIK CR00253873) **A** habitus, dorsal view **B** habitus, lateral view; Paratype (MABIK CR00253868) **C** rostrum. Scale bars are in μm.

***Habitus*** (Fig. 1A, B) subcylindrical, gradually tapering towards posterior caudal rami, with unclear separation between prosome and urosome. ***Prosome*** (Fig. 1A, B) slightly longer than urosome, comprising cephalothorax with completely fused first pedigerous somite, and three free pedigerous somites; posterior margins finely serrated. Cephalothorax bell-shaped, slightly longer than wide, ~ 28% of body length; integument covered with several sensilla, numerous minute pits and striped pattern (discernible under high resolution); rim with anastomosing patterns. P2-bearing somite longer than following two prosomites, with well-developed pleural area. Pleural areas of P3- and P4-bearing somites with pointed posterolateral corners.

***Urosome*** (Figs 1A, B, 3D) comprising P5-bearing somite, genital double-somite, and three free abdominal somites; integument of tergites covered with minute spinules and posterior margins finely serrated except for anal somite. Genital somite and third urosomite separated dorsally and laterally, but completely fused ventrally, forming genital double-somite (Figs 1A, B, 3D), with one pair of hook-like lateral projections on original genital somite; ventral surface with striations. Genital apertures (Fig. 3D) located far anteriorly, closed off by a single plate, on both sides with an outer vestigial seta and an inner bare seta, representing P6 (Fig. 4E; based on holotype). Copulatory pore located in median depression at level of gonopores. Anal somite (Figs 1A, 3D) as long as two preceding urosomites combined, ~ 2× as long as the caudal rami, with one pair of sensilla; semicircular operculum ornamented with minute spinules; anal sinus wide.

***Caudal rami*** (Figs 1A, B, 3D) cylindrical, ~ 1.7× as long as wide; surface covered with fine spinules; with one pore on ventro-distal surface; with seven setae: ventro-lateral seta I pinnate, short, inserted in proximal third of ramus, dorso-lateral seta II pinnate, twice as long as seta I; seta III pinnate, arising from outer ventro-distal corner, as long as caudal ramus; terminal setae IV and V well-developed, with internal fracture plane proximally, both setae fused basally, of which seta V longest, ~ 4× as long as seta IV; seta VI shortest, spiniform, located on ventro-posterior margin; tri-articulate seta VII plumose, issuing from dorsal surface subdistally.

***Rostrum*** (Fig. 1A, C) completely fused to cephalothorax, triangular, subdistally with paired sensilla and one ventral tube pore; anterior tip slightly concave.

**Figure 2.**
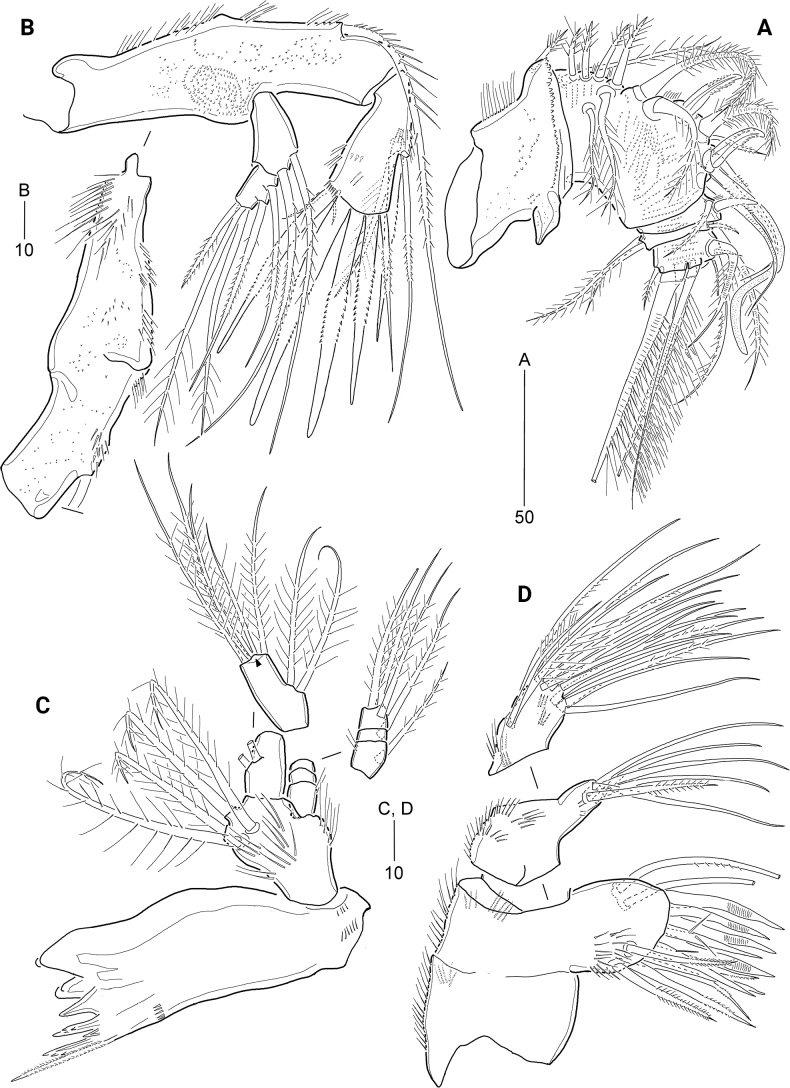
*Cerviniellabisegmenta* sp. nov. Female. Paratype (MABIK CR00253868) **A** antennule **B** antenna **C** mandible **D** maxillule. Scale bars are in μm.

***Antennule*** (Fig. 2A) robust, short, five-segmented, covered with diminutive dots (or denticles) as shown in Fig. 2A. First segment with a blunt conical process on outer distal corner. Second segment longest, with a distal peduncle bearing an aesthetasc fused to a seta. Forth segment shortest. Fifth segment distally with a small aesthetasc fused to a pinnate seta. Armature formula: 1 - [1 pinnate], 2 - [12 pinnate + 2 pinnate spine + (1 pinnate + ae)], 3-[3 pinnate], 4-[3 pinnate], 5-[6 pinnate + (1 pinnate + ae)].

***Antenna*** (Fig. 2B) three-segmented, comprising coxa, allobasis, and one-segmented endopod. Coxa small (not figured). Allobasis elongated, covered with various-sized spinules on surface, and with long spinules along abexopodal margin and a patch of denticles near base of exopod as shown in Fig. 2B; with one subdistal abexopodal seta. Endopod distinctly shorter than allobasis, with long spinules on inner and outer margins and rows of minute spinules along inner distal margin; lateral armature comprising one plumose and two pinnate elements; distal armature consisting of four serrate spines and three setae, of which outermost spine fused to one seta. Exopod two-segmented, proximal segment longer than distal segment, with two setae; distal segment with two lateral and three apical setae.

***Mandible*** (Fig. 2C). Coxa proximally with rows of spinules; gnathobase well-developed, with several multi-cuspidate teeth and one pinnate seta; outermost tooth largest; with two rows of minute spinules proximally and one row of spinules subdistally. Palp biramous, consisting of basis, three-segmented exopod, and one-segmented endopod; basis with four plumose setae distally, covered with spinules on surface, and with one row of setules on lateral margin. Exopod smaller than endopod; exp-1 and exp-2 each with one plumose lateral seta, and exp-3 with three plumose (one lateral and two apical) setae. Endopod with two lateral and five apical plumose setae, of which two apical setae fused at base (indicated by arrowhead in Fig. 2C).

**Figure 3.**
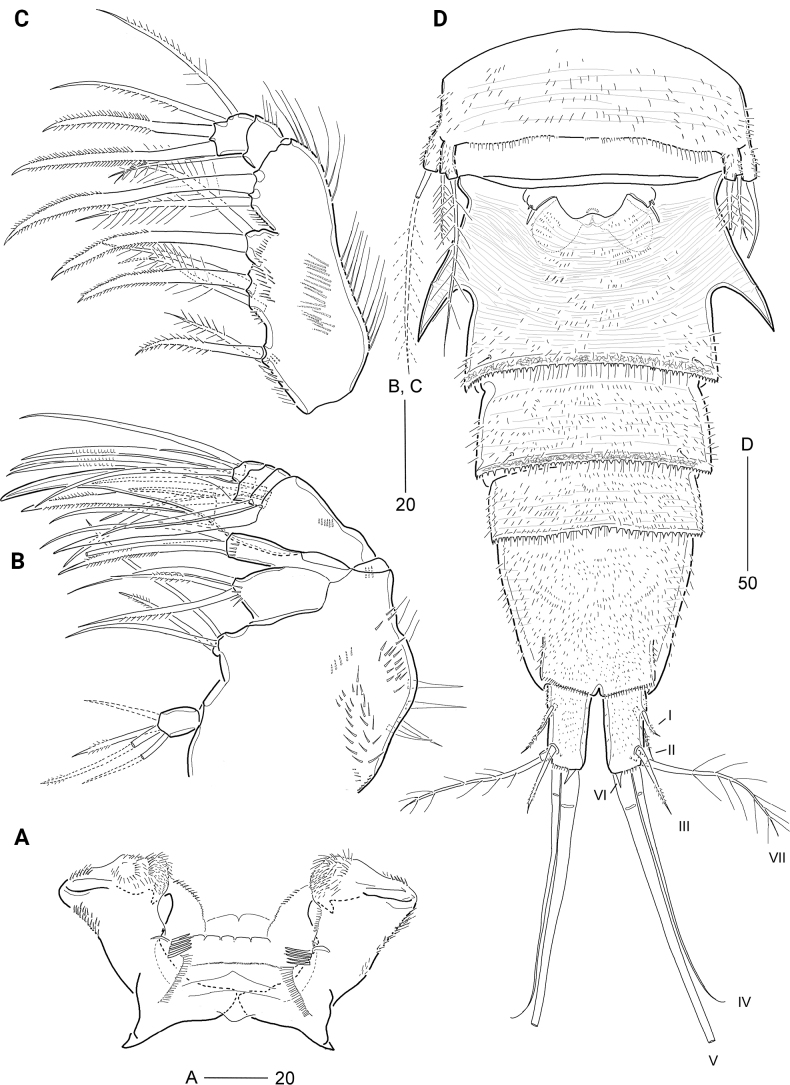
*Cerviniellabisegmenta* sp. nov. Female. Paratype (MABIK CR00253868) **A** paragnaths, anterior **B** maxilla **C** maxilliped **D** urosome, ventral view. Scale bars are in μm.

***Paragnaths*** (Fig. 3A) with well-developed chitinized lobes; distal and lateral margins covered with numerous spinules; posterior face with five strong spinules and one row of tiny spinules.

***Maxillule*** (Fig. 2D). Praecoxa ornamented with outer and subdistal spinules; arthrite well-developed, with two juxtaposed anterior setae, ten distal elements, and two pinnate posterior setae; posterior surface with patch of spinules. Coxa ornamented with outer spinules; cylindrical endite with one pinnate and five bare setae. Basis and endopod fused, with spinules on anterior and posterior surface; distal margin with 13 setae. Exopod represented by three pinnate setae.

***Maxilla*** (Fig. 3B). Syncoxa (damaged) large, with one row of stout spinules and one row of setules along outer margin, and five groups of spinules on anterior and posterior surfaces; with four endites: proximal praecoxal endite one-segmented, with two pinnate and two bare setae distally; distal praecoxal endite small, incorporated basally into syncoxa, with one pinnate and two bare setae distally; proximal coxal endite large, with one row of spinules subdistally, and one bare and two pinnate setae distally; distal coxal endite cylindrical, with one row of spinules subdistally, and one pinnate spine and two setae (one bare and one pinnate) distally. Allobasis large, with one stout spine and two setae distally; inner part drawn out into a curved strong claw accompanied by one stout pinnate spine and two setae. Endopod small, three-segmented; first segment with two bare setae; second segment with one bare and one geniculate seta; distal segment with one geniculate and three bare setae.

***Maxilliped*** (Fig. 3C) three-segmented, composed of protopod, and two-segmented endopod. Protopod elongate, with two rows of long outer setules, one row of posterior spinules; with four endites ornamented with inner and anterior spinules: three syncoxal endites represented proximal to distal by one pinnate spine and one pinnate seta, one pinnate spine and two plumose setae, and one pinnate spine and one plumose seta; basal endite represented by one pinnate spine and one plumose seta. Endopod small; first segment with one row of outer spinules and one plumose subdistal seta; second segment with two pinnate distal spines and two pinnate lateral setae, of which proximal one plumose proximally.

**Figure 4.**
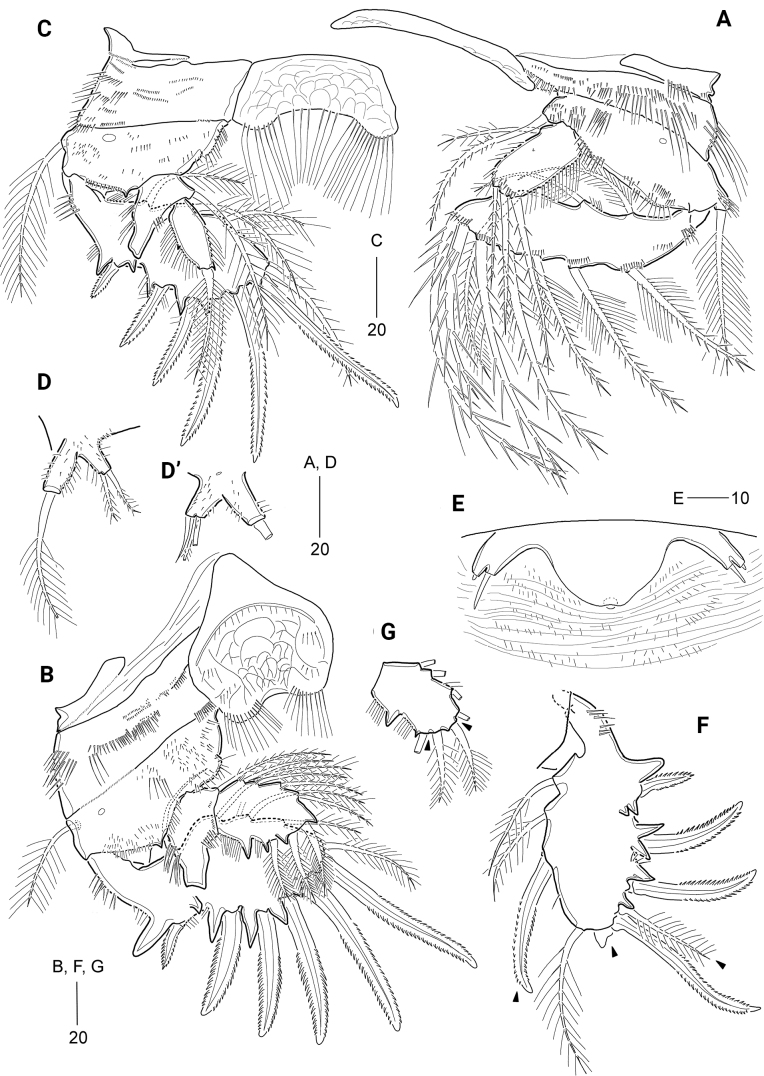
*Cerviniellabisegmenta* sp. nov. Female. Paratype (MABIK CR00253868) **A**P1, anterior **B**P2, anterior **C**P3, anterior **D**P4, anterior **D**’ P4, showing abnormal setae on the other pair; Holotype (MABIK CR00253873) **E**P6; Paratype (MABIK CR00253869) **F**P2exp**G**P2enp-2. Scale bars are in μm.

***P1*** (Fig. 4A). Intercoxal sclerite transversely elongated and narrow, with surface reticulation distally. Praecoxa with spinules on anterior surface. Coxa with various-sized spinules and setules on anterior surface. Basis larger than coxa, with one anterior pore and several rows of anterior spinules; with one long plumose outer and one pinnate inner seta; rami set far away from each other. Exopod one-segmented, with a row of setules on inner margin, and rows of spinules on outer proximal margin and around bases of outer setae; with three inner, two apical, and five outer setae. Endopod one-segmented, with one anterior pore medially, setules along inner and outer margins, and anterior spinules around distal margin; with one inner, one outer, and two apical setae.

***P2*** (Fig. 4B) larger than other thoracic legs. Intercoxal sclerite well-developed, cordiform; anterior surface with conspicuous reticulation, distally with one pair of rows of long setules; distal margin concave. Praecoxa small, with minute spinules along distal margin. Coxa with several rows of various-sized anterior spinules and three rows of long outer spinules. Basis with one anterior pore and numerous rows of various-sized anterior spinules; distal margin with one small acute process between rami; plumose outer seta shorter than those of P1 and P3. Exopod one-segmented, with one anterior pore distally and several rows of setules along inner and outer margins; with five serrate outer spines, increasing in size distally, one serrate apical spines, one plumose apical seta, and four plumose inner setae; outer and distal margins with four acute and three small processes near base of spines. Endopod two-segmented, shorter than exopod; enp-1 with three outer rows of setules and two distal rows of minute spinules, outer margin drawn out into a chitinous process bearing weakly concave tip and inner margin with one plumose seta; enp-2 longer than preceding one, ~ 1.6× as long as wide, with one outer, two apical, and four inner pinnate setae, outer and distal margins with two acute processes.

***P3*** (Fig. 4C) smaller than P2. Intercoxal sclerite well-developed, subrectangular, with surface reticulation; distal margin concave, with long setules. Praecoxa small, with two distal rows of minute spinules. Coxa with numerous rows of various-sized anterior spinules. Basis with numerous minute anterior spinules and row of inner setules, and one small acute process between rami; anterior pore larger than that of P2; outer seta plumose and ~ 3× as long as outer margin of basis. Exopod one-segmented, with one row of outer spinules and several rows of outer and inner setules; outer margin with two blunt and five acute processes; with five serrate outer spines, increasing in size distally, one serrate apical spine and one pinnate apical seta, and three plumose inner setae. Endopod two-segmented, distinctly shorter than exopod; enp-1 with three groups of outer spinules and one plumose inner seta, outer corner drawn out into a chitinous blunt process; enp-2 oval, ~ 2.3× as long as wide, with one plumose apical seta, outer and inner margins with row of setules.

***P4*** (Fig. 4D) rudimentary, uniramous, comprising outer setophore and exopod, fused basally to fourth pedigerous somite, covered with various-sized spinules; outer setophore cylindrical, longer than exopod, with long plumose seta. Exopod one-segmented, with one apical and one outer setae. Endopod absent.

Armature formulae of P1–P4 as follows:

**Table:** 

Leg	Exopod	Endopod
P1	325	121
P2	425	1.[2–4]21
P3	325	1.010
P4	011	absent

***P5*** (Fig. 3D) bilobate as in P4, fused to somite, covered with minute spinules; outer setophore cylindrical, with one plumose seta. Exopod slightly exceeding setophore, with one plumose outer and one plumose apical seta, the latter ~ 2.5× longer than outer seta.

**Male.** Unknown.

#### Variability.

The morphological variation in *Cerviniellabisegmenta* sp. nov. appears in the armature formula of thoracopods. P2enp-2 presumably has four inner setae in the normal condition, but the P2enp-2 of some specimens (four of 18 specimens) has two or three inner setae.

#### Abnormality.

Abnormal exopod and endopod of P2 were observed in one specimen (paratype, MABIK CR00253869) (Fig. 4F, G). In comparison with the normal condition of P2 (Fig. 4B), the abnormal exopod has a seta in place of the proximal fourth outer spine and a spine in place of the proximal third inner seta (Fig. 4F, indicated by arrowheads), and the enp-2 lacks a small pointed protrusion on either side of the two apical setae (Fig. 4G, indicated by arrowheads). In addition, paratype (MABIK CR00253868) displayed two setae (the normal condition) on one of the exopods of P4 (Fig. 4D) and three setae on the other exopod (Fig. 4D’).

#### Etymology.

The specific name *bisegmenta* is derived from a combination of the Latin prefix *bi*-, meaning ‘having two parts’ and the Latin noun *segmentum*, meaning ‘cutting’ or ‘piece’, and refers to the two-segmented antennary exopod, which is an autapomorphy of this species. It is a noun in the nominative plural.

#### Remarks.

Based on the presence or absence of the P4 endopod, Apostolov (2011) subdivided the genus *Cerviniella* into the *brodskayae* and *mirabilipes* groups. *Cerviniellabisegmenta* sp. nov. lacks the ramus and can be classified within the *mirabilipes* group, which includes *C.danae*, *C.lagarderei*, *C.langi*, *C.mirabilipes*, and *C.talpa*.

The segmentation of the female antennule is useful for differentiating among species of *Cerviniella*. *Cerviniellabisegmenta* sp. nov. has an advanced five-segmented antennule, shared in *C.danae* and *C.hitoshii*, but the latter species is in the *brodskayae* group. The other 11 *Cerviniella* species have a six- or seven-segmented antennule (see discussion below).

*Cerviniellabisegmenta* sp. nov. can be easily distinguished differs in several characters from the same group, *C.danae*. First, the antenna of *C.bisegmenta* sp. nov. has a two-segmented exopod, compared with the four-segmented antennary exopod of *C.danae*. Second, the mandibular endopod of *C.bisegmenta* sp. nov. has two lateral setae, whereas the mandibular endopod of *C.danae* has three lateral setae. Third, the basis and endopod of the maxillule of *C.bisegmenta* sp. nov. have a total of 13 setae, compared with 14 setae in *C.danae*. Fourth, the syncoxa of the maxilliped of *C.bisegmenta* sp. nov. has nine setae and spines, compared with the seven elements of *C.danae*. Fifth, the P1 endopod of *C.bisegmenta* sp. nov. has four setae, compared with six setae in *C.danae*. Sixth, the P3 exopod of *C.bisegmenta* sp. nov. has three inner setae, compared with two inner setae in *C.danae*. Finally, the caudal ramus of *C.bisegmenta* sp. nov. is short with a 1.7-fold length relative to width, compared with a 3.5-fold length for *C.danae*. The first and seventh aforementioned characteristics provide conclusive evidence for the identification of *C.bisegmenta* sp. nov. In all species of *Cerviniella*, except *C.bisegmenta* sp. nov., the antennary exopod is four-segmented, and the length of the caudal rami is > 3-fold to width and longer than the length of the anal somite.

### ﻿ Cerviniella permixta sp. nov.

Taxon classificationAnimaliaHarpacticoidaAegisthidae

BEA18994-5170-5E03-A7CE-FD2A9712A640

https://zoobank.org/1F5C528D-CAE9-432C-838B-2714621D45BE

Figs 5 , 6 , 7 , 8

#### Type locality.

The Yellow Sea; 35°00'05.44"N, 125°59'44.49"E; 88.1 m depth.

#### Type material.

***Holotype*.** One ♀ preserved in a vial with 80% ethanol (MABIK CR00253875); collected from the type locality, March 02, 2022. ***Paratypes*.** One ♀ (MABIK CR00253872) dissected on 10 slides, two ♀♀ (MABIK CR00253876) preserved in a vial with 80% ethanol, collection data as in holotype; J.G. Kim leg.

#### Description.

**Female** (based on the holotype and paratypes). ***Body*** length from anterior margin of rostrum to posterior margin of caudal rami (paratype, MABIK CR00253872, in lateral view, telescoping of somites not considered) 857 μm (range: 857–886 μm, *n* = 4, holotype: 861 μm).

**Figure 5.**
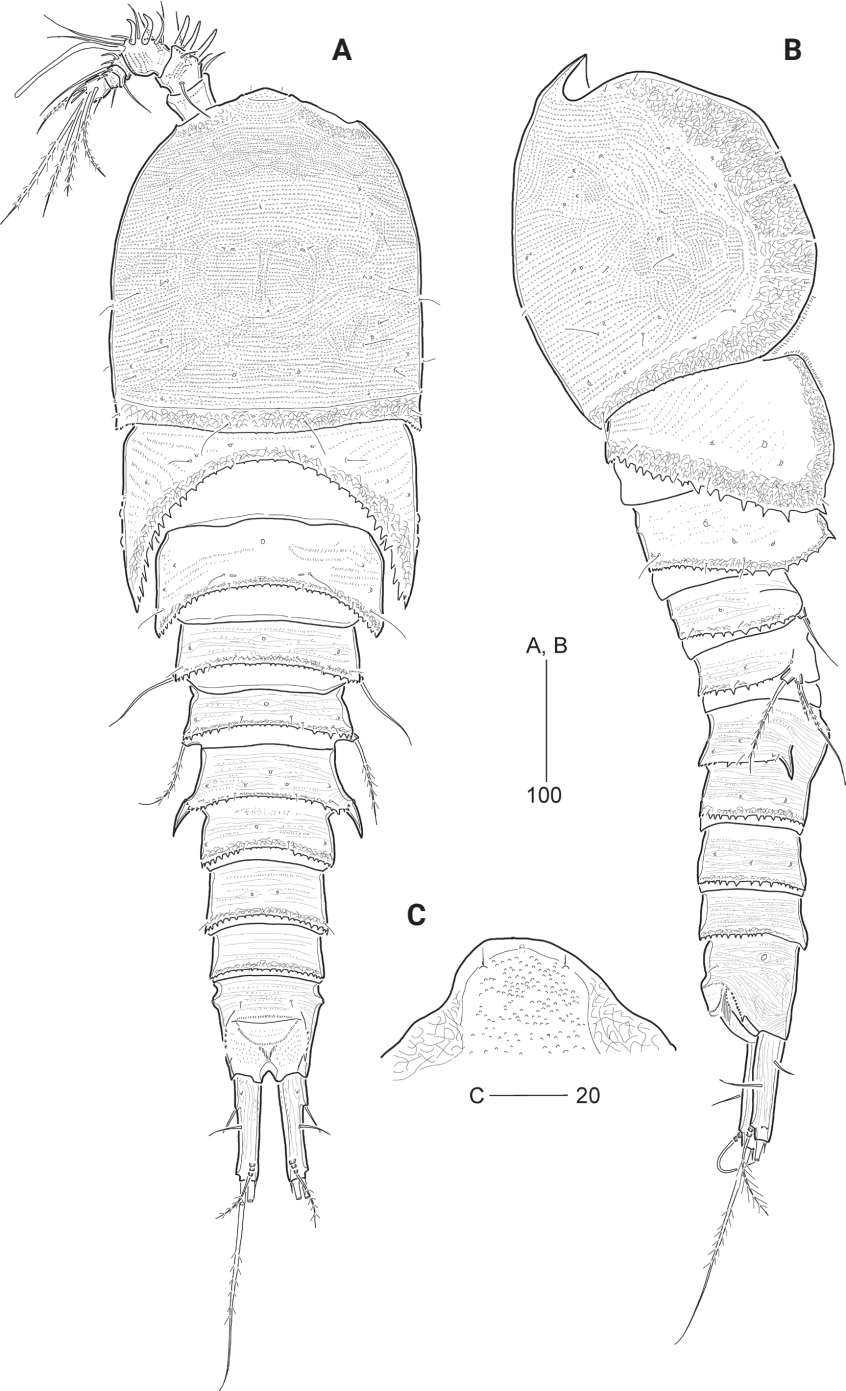
*Cerviniellapermixta* sp. nov. Female. Holotype (MABIK CR00253875) **A** habitus, dorsal view **B** habitus, lateral view; Paratype (MABIK CR00253872) **C** rostrum. Scale bars are in μm.

***Habitus*** (Fig. 5A, B) subcylindrical, gradually tapering posteriorly, with unclear separation between prosome and urosome.

***Prosome*** (Fig. 5A, B) slightly longer than urosome, comprising cephalothorax (with completely fused first pedigerous somite) and three free pedigerous somites. Cephalothorax bell-shaped, slightly longer than wide, ~ 31% of body length; surface embossed with numerous minute dots (or denticles) and striped pattern (discernible under high resolution), covered with several sensilla and pores; rim with anastomosing patterns; ventro-posterior margins with row of fine spinules and posterior margin weakly serrate. Pedigerous somites bearing P2–P4 with several sensilla, minute spinules and pores as shown in Fig. 5A, B. Pleural areas of P2-bearing somite more extended than those of other prosomites, P2- and P3-bearing somites with pointed posterolateral corners; posterior margins distinctly serrate.

**Figure 6.**
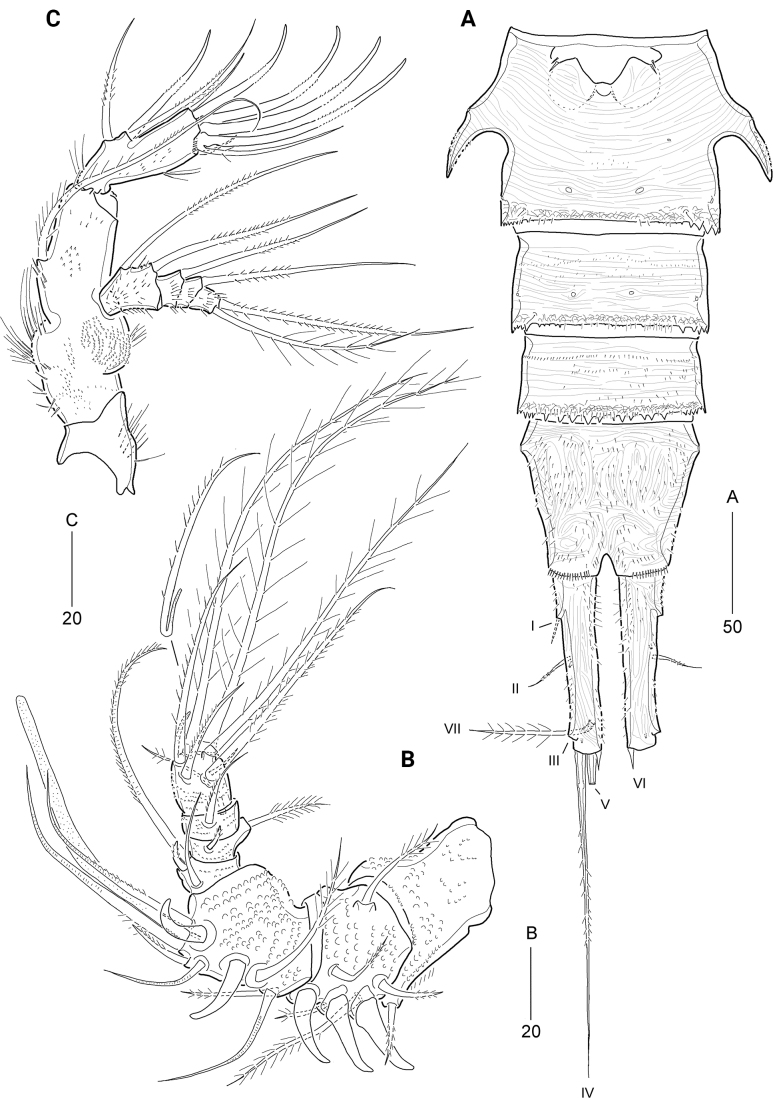
*Cerviniellapermixta* sp. nov. Female. Paratype (MABIK CR00253872) **A** urosome **B** antennule **C** antenna. Scale bars are in μm.

***Urosome*** (Figs 5A, B, 6A) comprising P5-bearing somite, genital double-somite, and three free abdominal somites; surface covered with striation and minute spinules (or denticles); posterior margins finely serrated except for anal somite, pore pattern on dorsal surface as indicated in Fig. 5A. Genital somite and third urosomite separated dorsally and laterally, but completely fused ventrally forming genital double-somite (Figs 5A, B, 6A), with one pair of hook-like lateral projections on original genital somite. Genital apertures (Fig. 6A) located far anteriorly; closed off by single plate, on both sides with an outer vestigial seta and an inner bare seta, representing P6 (Fig. 6A). Copulatory pore located in the middle. Anal somite (Figs 5A, B, 6A) slightly shorter than two preceding urosomites combined; as long as caudal rami; with one pair of sensilla dorsally and one pair of pores laterally; with semicircular operculum ornamented with minute spinules; lateral margin of anal opening with spinules row and a spinous process.

***Caudal rami*** (Figs 5A, B, 6A) cylindrical, ~ 4× as long as greatest width, surface covered with stripes and fine spinules; with pairs of pores on dorsal and ventral surface; with seven setae: ventro-lateral seta I bare, short, inserted in proximal third of ramus, dorso-lateral seta II pinnate, ~ 1.5× longer than seta I; seta III missing (this seta missing in all specimens), arising from outer subdistal corner; terminal setae IV and V well-developed, with internal fracture plane proximally, both setae fused basally, seta V longest (but damaged in all specimens); seta VI shortest, spiniform, located on ventro-posterior margin; tri-articulate seta VII plumose, issuing from dorsal surface subdistally.

***Rostrum*** (Fig. 5A, C) completely fused to cephalothorax, triangular, subdistally with one pair of sensilla and one ventral tube pore; apical tip blunt.

***Antennule*** (Fig. 6B) robust, short, seven-segmented, covered with numerous diminutive dots (or denticles) as shown in Fig. 6B. First segment largest. Third segment with one peduncle bearing a seta fused to an aesthetasc. Fourth to sixth segments small. Seventh segment subdistally with a pinnate seta fused to an aesthetasc. Armature formula: 1-[1 pinnate], 2-[5 pinnate + 3 spine], 3-[2 + 4 pinnate + 2 spine + (1 pinnate + ae)], 4-[1 + 1 pinnate + 1 pinnate spine], 5-[1 pinnate + 1 pinnate spine], 6-[2 pinnate], 7-[1+ 5 pinnate + (1 pinnate + ae)].

***Antenna*** (Fig. 6C) three-segmented, comprising coxa, allobasis, and one-segmented endopod. Coxa small, with long outer spinules. Allobasis elongate, covered with several group of minute spinules, outer margin with one group of setules, and inner margin with two rows of long setules and two rows of spinules; area close to the insertion of exopod swollen; with one subdistal abexopodal seta. Endopod distinctly shorter than allobasis, with one row of outer setules, one row of inner setules, and one row of minute distal spinules; lateral armature composed of one serrate spine and two pinnate setae; distal armature consisting of four serrate spines, one pinnate seta, one plumose seta, and one bare seta; outermost serrate spine fused basally to neighboring seta. Exopod four-segmented, covered with minute spinules; proximal segment longer than other segments, with two pinnate lateral setae; second and third segments each with one pinnate lateral seta; distal segment distally with one pinnate and one plumose seta.

**Figure 7.**
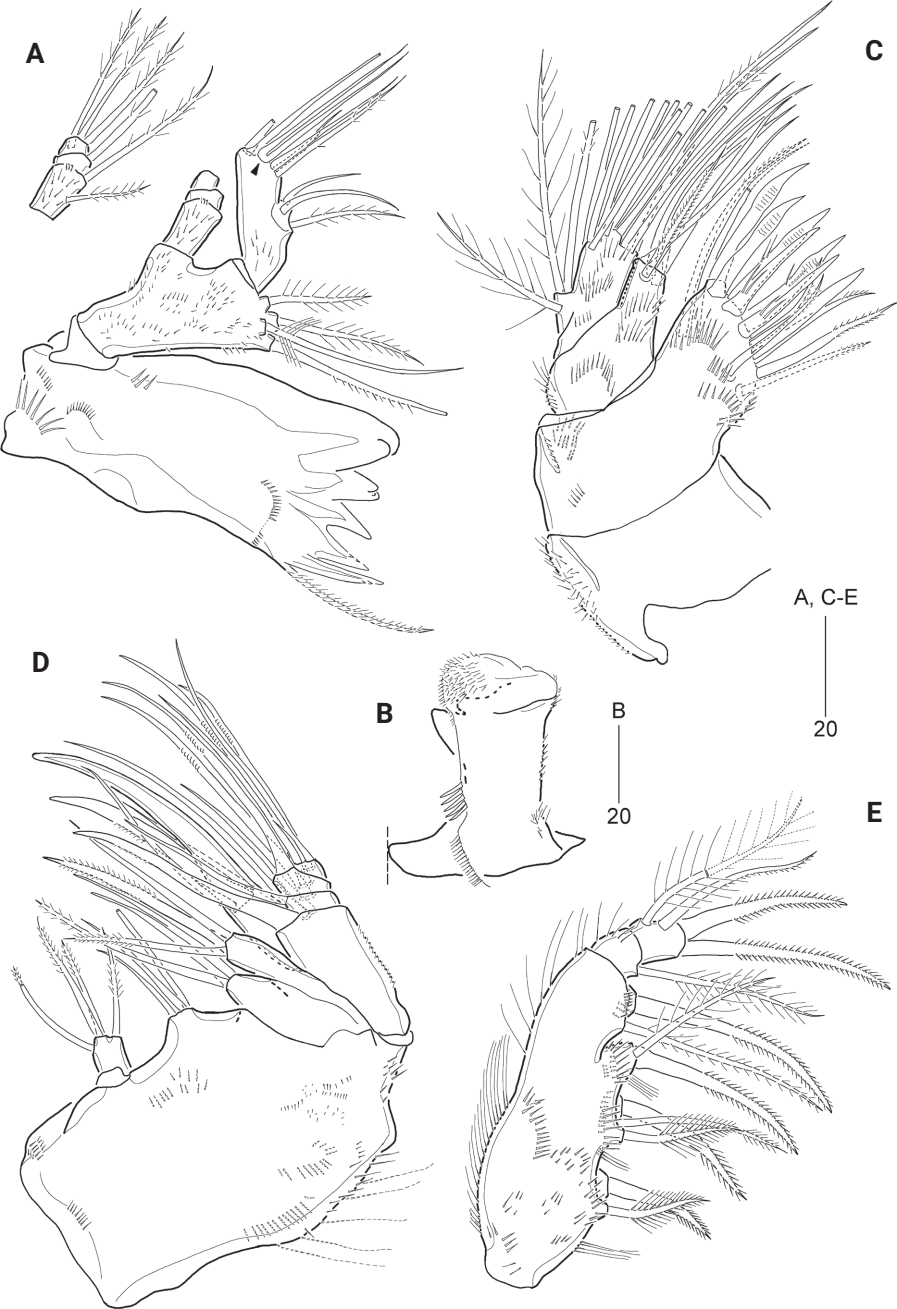
*Cerviniellapermixta* sp. nov. Female. Paratype (MABIK CR00253872) **A** mandible **B** paragnaths, anterior, one side **C** maxillule **D** maxilla **E** maxilliped. Scale bars are in μm.

***Mandible*** (Fig. 7A). Coxa well-developed, proximally with four rows of various-sized spinules and subdistally with one row of minute spinules; gnathobase well-developed, with one uni-cuspidate and four multi-cuspidate teeth, of which outermost tooth largest, and one pinnate seta. Palp biramous, consisting of basis, three-segmented exopod, and one-segmented endopod; basis covered with minute spinules, subdistally with one pinnate and three plumose setae, of which subdistal one distally bare and proximally plumose. Exopod shorter than endopod; exp-1 longer than other two distal segments combined, covered with minute spinules, with one small plumose seta laterally and one long plumose seta subdistally; exp-2 small, with one pinnate seta; exp-3 small, with three plumose setae distally. Endopod 1.4× longer than exopod, laterally with one pinnate and two bare setae, and distally with one pinnate and six bare setae, of which two bare setae fused at base (indicated by arrowhead in Fig. 7A).

***Paragnaths*** (Fig. 7B) (damaged during dissection process, one side figured) with well-developed chitinized lobes; distal margin with numerous spinules; posterior face with five strong spinules and one row of tiny spinules; lateral margin with tiny spinules.

***Maxillule*** (Fig. 7C). Praecoxa ornamented proximally with several rows of outer spinules and subdistally with three rows of anterior spinules and two rows of posterior spinules; arthrite well-developed, with two juxtaposed pinnate setae anteriorly (one missing seta indicated by the dotted line in Fig. 7C), seven spines and three pinnate setae distally, and two pinnate setae posteriorly (one missing seta indicated by the dotted line in Fig. 7C); posterior surface with several rows of spinules. Coxa with one row of outer spinules and three rows of posterior spinules; endite cylindrical, with one pinnate and five bare setae subdistally or distally. Basis broad, with several rows of posterior spinules and ten distal setae. Exopod represented by one pinnate and two plumose setae. Endopod completely fused basally to basis, represented by three setae.

***Maxilla*** (Fig. 7D). Syncoxa large, with numerous rows of spinules anteriorly and posteriorly and one row of long outer setules; with four endites: proximal praecoxal endite separated basally from syncoxa, with four pinnate setae distally; distal praecoxal endite rudimentary, represented by three bare setae; proximal coxal endite cylindrical, with three bare setae distally; distal coxal endite also cylindrical, with one pinnate spine and two setae distally (one bare and one pinnate). Allobasis with one unipinnate stout spine and two bare setae distally; inner part drawn out into a curved strong claw accompanied by one unipinnate stout spine and two bare setae. Endopod small, three-segmented; first segment with one bare and one geniculate seta; second segment with two geniculate setae; distal segment with one geniculate and three bare setae.

***Maxilliped*** (Fig. 7E) three-segmented. Protopod elongate, with two and three rows of long setules along outer and inner margins and numerous rows of spinules on anterior and posterior surfaces; with four endites: syncoxal endites represented proximal to distal by two pinnate spines, three pinnate spines, and one pinnate spine and one plumose seta; basal endite represented by one pinnate spine and one plumose seta. Endopod two-segmented, with one row of outer setules on first segment, the latter with one plumose seta; second segment with two pinnate spines distally, and one unipinnate and one plumose seta laterally.

**Figure 8.**
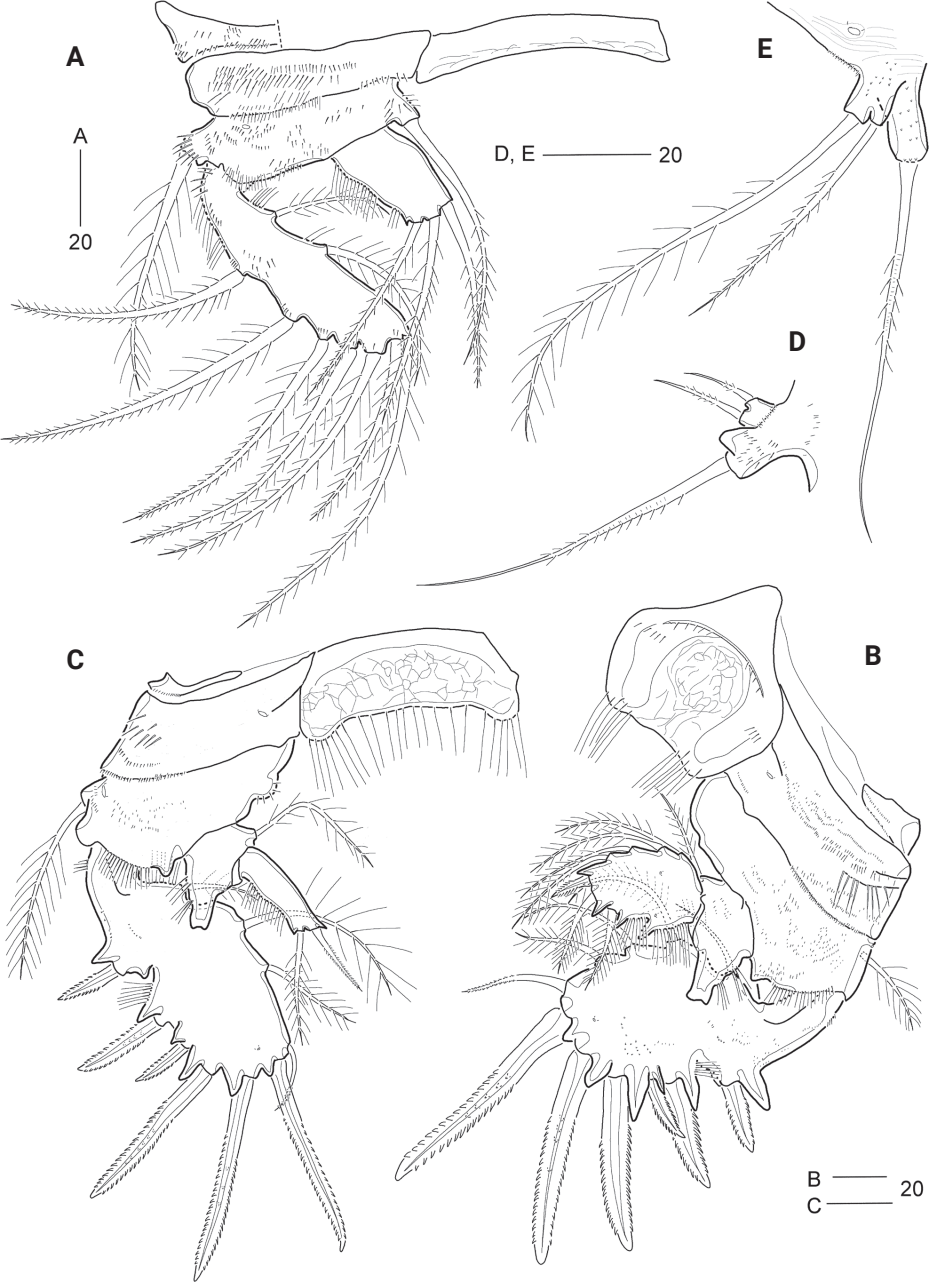
*Cerviniellapermixta* sp. nov. Female. Paratype (MABIK CR00253872) **A**P1, anterior **B**P2, anterior **C**P3, anterior **D**P4**E**P5. Scale bars are in μm.

***P1*** (Fig. 8A). Intercoxal sclerite transversely elongated and narrow, with weak surface reticulation distally. Praecoxa (damaged) small, with anterior and distal spinules. Coxa wide, with various-sized spinules anteriorly and distally. Basis wide, somewhat larger than coxa, with one anterior pore and several rows of spinules anteriorly and distally; outer setophore with one row of anterior spinules and one plumose seta; inner distal corner produced, with one pinnate seta slightly exceeding end of exopod. Exopod one-segmented, elongate, with rows of outer and inner setules and two groups of anterior spinules, and several groups of spinules at bases of outer and distal setae; with two plumose inner, two pinnate apical, and five pinnate outer setae. Endopod one-segmented, ~ 1/2 exopod length, with two rows of outer setules and one outer and two apical setae.

***P2*** (Fig. 8B) larger than other legs, anterior surface covered with striated patterns (not figured). Intercoxal sclerite well-developed, cordiform, slightly concave distally, with surface reticulation, two pairs of anterior spinular rows and one pair of distal setular rows. Praecoxa small, triangular, with one row of minute spinules anteriorly and one row of minute spinules distally. Coxa large, wide; anterior surface with one pore, one row of large spinules and numerous rows of minute spinules; distal margin with one row of minute spinules. Basis large, with numerous rows or groups of fine spinules anteriorly and one row of setules distally; distal margin with small process between rami, and outer margin with one pore and one plumose seta. Exopod one-segmented, expanded distally; with five serrate outer spines, of which proximal third one shortest, one serrate apical spine, one pinnate apical seta, and four plumose inner setae; outer and distal margins with one blunt and five acute processes at bases of spines; anterior surface with one small pore and several rows of spinules; outer margin with one row of fine spinules and one row of setules; posterior surface with several rows of spinules (not figured). Endopod two-segmented, reaching distal fourth of exopod; enp-1 with several minute anterior spinules, two rows of outer setules, one plumose inner seta and one distal pore; outer corner drawn out into a chitinous process with weakly bifid tip; enp-2 with one plumose outer seta, one plumose apical seta, one small pinnate apical spine, and four plumose inner setae, and two anterior pores; distal and outer margins each with two acute processes.

***P3*** (Fig. 8C) smaller than P2; anterior surface covered with striated patterns (not figured). Intercoxal sclerite well-developed, subrectangular, with surface reticulation and one row of long distal setules. Praecoxa small, with one row of minute spinules distally. Coxa large, with one large pore, one group of spinules and one row of minute spinules on anterior surface, and one row of minute spinules along distal margin. Basis larger than coxa; anterior surface with one large pore and several rows of fine spinules; outer setophore with one long plumose seta; inner margin with one rounded protrusion bearing one row of setules; distal margin with one small process between rami and one row of setules. Exopod one-segmented, expanded, with five serrate outer spines, of which proximal third one shortest, one serrate apical spine, one pinnate apical seta, and three plumose inner setae; outer and distal margins with four acute and three small processes; anterior surface with several fine spinules and one small pore; posterior surface with several spinules (not figured); inner and outer margins with one and two rows of setules, respectively. Endopod two-segmented, slightly exceeding 1/2 of exopod length; enp-1 as in P2, with outer chitinous process, two rows of outer setules, and one plumose inner seta; enp-2 elongate, 3× as long as wide, with one row of outer setules, one pinnate apical seta, and one plumose outer seta.

***P4*** (Fig. 8D) visibly reduced, composed of outer setophore and exopod, fused basally to supporting somite. Outer setophore larger than exopod, covered with minute anterior spinules, with one long pinnate seta apically and one small inner protrusion subapically. Exopod one-segmented, small, with one pinnate apical and one pinnate outer seta. Endopod absent.

Armature formulae of P1–P4 as follows:

**Table:** 

Leg	Exopod	Endopod
P1	225	021
P2	425	1.421
P3	325	1.011
P4	011	absent

***P5*** (Fig. 8E) fused basally to supporting somite as in *C.bisegmenta* sp. nov. Outer setophore cylindrical, covered with small denticles, with one long pinnate seta. Exopod small, fused basally to baseoendopod, covered with small denticles, with one plumose apical and one pinnate outer seta; apical seta 1.8× longer than outer seta.

**Male.** Unknown.

#### Etymology.

The specific name *permixta* is derived from the Latin adjective *permixtus*, meaning ‘mixed’ or ‘confused’, and refers to the fact that several diagnostic characteristics of *Cerviniella* species are mixed in this species. It is in the nominative singular. Gender: feminine.

#### Remarks.

*Cerviniellapermixta* sp. nov. lacks a P4 endopod and is placed in the *mirabilipes* group as *C.bisegmenta* sp. nov. described above. This new species can be distinguished from *C.bisegmenta* sp. nov. on the basis of several morphological differences. *Cerviniellapermixta* sp. nov. is characterized by the presence of numerous minute pits on the cephalothorax surface, whereas *C.bisegmenta* sp. nov. is characterized by numerous minute dots or denticles. *Cerviniellapermixta* sp. nov. has a blunt rostral tip, whereas *C.bisegmenta* sp. nov. has a concave tip. The antennule has seven segments in *C.permixta* sp. nov. and five segments in *C.bisegmenta* sp. nov. The P1enp-1 lacks the inner seta and P3enp-2 has two setae in *C.permixta* sp. nov., whereas the P1enp-1 has an inner seta and P3enp-2 has one seta in *C.bisegmenta* sp. nov. The caudal ramus of *C.permixta* sp. nov. is relatively long, with a 4-fold length relative to width, compared with a 1.7-fold length for *C.bisegmenta* sp. nov.

In the *mirabilipes* group, the species with a seven-segmented antennule are *C.lagarderei*, *C.langi* (but illustrated as six-segmented in the original description (Bodin 1968: pl. III); see discussion below), *C.mirabilipes*, *C.talpa*, as well as *C.permixta* sp. nov. However, *C.permixta* sp. nov. is different from these four species in several characteristics. First, *C.permixta* sp. nov. has two setae on the distal exopodal segment of the antenna, compared with three setae in *C.langi*. Second, *C.permixta* sp. nov. has a three-segmented mandibular exopod, compared with a one-segmented mandibular exopod in *C.mirabilipes* and four-segmented in *C.lagarderei* and *C.langi*. Third, the P1enp-1 of *C.permixta* sp. nov. has three setae, compared with six in *C.lagarderei* and *C.talpa*, and seven in *C.langi*. Fourth, the P2enp-2 apical margin has a spine-like element in *C.permixta* sp. nov., compared with a seta-like element in the remaining four species. Fifth, the P3enp-2 of *C.permixta* sp. nov. has two setae, compared with four setae in *C.langi* and *C.talpa*, and three setae on the one-segmented P3 endopod in *C.lagarderei*. Finally, the reduced P4 exopod of *C.permixta* sp. nov. has two setae, compared with four setae in *C.talpa* and five setae in *C.mirabilipes*; *C.lagarderei* has the well-developed and two-segmented ramus with ten setae/spines.

### ﻿Key to species of the genus *Cerviniella*

A classification key for the 14 *Cerviniella* species (excluding *C.peruana*) is presented, including the two new species described from the Yellow Sea.

**Table:** 

1	Antennule 5-segmented	**2**
–	Antennule different	**4**
2	P4 endopod 2-segmented	***C.hitoshii* Kihara & Martínez Arbizu, 2012**
–	P4 without endopod	**3**
3	P1 endopod with 4 setae; P3 exopod with 10 setae/spines; antennary exopod 2-segmented; caudal rami < 2× as long as wide	***C.bisegmenta* sp. nov.**
–	P1 endopod with 6 setae; P3 exopod with 9 setae/spines; antennary exopod 4-segmented; caudal ramus 3.5× as long as wide	***C.danae* Kihara & Martínez Arbizu, 2012**
4	P1 endopod 2-segmented	**5**
–	P1 endopod 1-segmented	**6**
5	P2 exopod with 10 setae/spines; caudal ramus 6.2× as long as wide	***C.bodini* Coull, 1973**
–	P2 exopod with 11 setae/spines; caudal ramus 4.3× as long as wide	***C.abyssalis* Apostolov, 2011**
6	P4 exopod 2-segmented	***C.lagarderei* Bodin, 1968**
–	P4 exopod 1-segmented	**7**
7	P3 endopod 1-segmented	**8**
–	P3 endopod 2-segmented	**9**
8	P3 endopod with 2 setae; P3 exopod with 8 setae/spines	***C.longifurcata* Apostolov, 2011**
–	P3 endopod with 3 setae; P3 exopod with 11 setae/spines	***C.brodskayae* Por, 1969**
–	P3 endopod with 4 setae; P3 exopod with 9 setae/spines	***C.hamata* Coull, 1973**
9	P2enp-1 without inner seta; P2 exopod with 10 setae/spines	***C.talpa* (Por, 1964)**
–	P2enp-1 with 1 inner seta; P2 exopod with 11 setae/spines	**10**
10	P1 exopod with 10 setae/spines; P3 exopod with 11 setae/spines	***C.langi* Bodin, 1968**
–	P1 exopod with 9 setae/spines; P3 exopod with 10 setae/spines	**11**
11	P4 exopod with 2 setae/spines; P5 exopod with 2 setae; P1 endopod with 3 setae	***C.permixta* sp. nov.**
–	P4 exopod with 4 setae/spines; P5 exopod with 3 setae; P1 endopod with 4 setae	***C.arctica* Kihara & Martínez Arbizu, 2012**
–	P4 exopod with 5 setae/spines; P5 exopod with 3 setae; P1 endopod with 3 setae	***C.mirabilipes* Smirnov, 1946**

## ﻿Discussion

Recently, Khodami et al. (2020) proposed a new subfamily Cerviniellinae, which includes the genera *Cerviniella* (type genus), *Eucanuella*, and *Hase*. The subfamily was defined on the basis of short female antennules with modified spines, hook-like lateral projections on the urosome, and inward-bent P1 exopods (Khodami et al. 2020). The subfamily members adapt to benthic habitats with spade-like appendages specialized for burrowing, short female antennules with strong spines, inward-bent exopods with strong outer spines and reduced endopods of thoracic legs, and short caudal rami. Similar adaptations are observed in some genera of benthic harpacticoid families, e.g., Cletodidae Scott, T., 1905, Nannopodidae Brady, 1880, and Paramesochridae Lang, 1944. The morphologies of the genera *Cerviniella* and *Hase* are linked to their inbenthic lifestyles. However, the morphology of thoracic legs and male caudal rami in the genus *Eucanuella* suggests an epibenthic or hyperbenthic lifestyle, particularly in males, contrary to the suggestion of Khodami et al. (2020). Similar to members of other subfamilies (e.g., Cerviniinae and Cerviniopseinae), the rami of thoracic legs in *Eucanuella* species are well-developed and three-segmented, and the male caudal rami are elongated and parallel.

Khodami et al. (2020) analyzed the molecular evidence and suggested that the family Aegisthidae evolved from an undescribed benthic taxon, Aegisthidae gen. 1, which retained ancestral features (e.g., incomplete fusion of the first thoracic somite into the cephalosome and three-segmented rami of the thoracic legs). In contrast, the subfamily Cerviniellinae, a sister taxon of Aegisthidae gen. 1, has independently colonized deep-sea benthic habitats, whereas other subfamilies have colonized different habitats (e.g., epibenthic, hyperbenthic, planktonic, and associated habitats) (cf. Khodami et al. 2020: fig. 7). As mentioned previously, the genus *Eucanuella* has intermediate features between Aegisthidae gen. 1 and *Cerviniella*/*Hase*, suggesting that it is the basal taxon within the subfamily Cerviniellinae.

Kihara and Martínez Arbizu (2012) revised the diagnostic criteria for the genus *Cerviniella*. However, they were not aware of the study of Apostolov (2011). In the revised diagnostic criteria of Kihara and Martínez Arbizu (2012), the genus is morphologically characterized by female antennules with 5–7 segments with robust modified elements, hook-like lateral projections on genital double-somites, inward-bent single segment of P1–P4 exopods (except for *C.lagarderei*), absent or one or two segments on P1–P4 endopods, and P5 endopodal lobe absent (Kihara and Martínez Arbizu 2012). The second and third characteristics are the main differentiating features between *Cerviniella* and the other two genera (Smirnov 1946; Kihara and Martínez Arbizu 2012; Corgosinho et al. 2018). Because the two newly described Korean species, *C.bisegmenta* sp. nov. and *C.permixta* sp. nov., exhibit these features, they are classified within the genus *Cerviniella*.

Becker (1974) found one female specimen of the deep-sea species *C.peruana* from the Southwestern Pacific region (Peru) at a depth of 5,000 m. Based on its particularly one-segmented P1–P4 exopods, and the presence of an outer protrusion on P2–P4enp-1, the species was attributed to the genus *Cerviniella*. However, the accompanying illustration of the female habitus showed nine body somites (see Becker 1974: abb. 1), suggesting that an immature specimen of this deep-sea species had been examined. Adult females of *Cerviniella* species have ten body somites, including the cephalothorax and genital double-somites, which are separated dorsally and fused ventrally. The characteristics of the immature copepods of this species are unclear (Fiers 1997; Ivanenko et al. 2008; Björnberg 2010; Menzel 2011). For example, the adults and copepodid V of *Haselagomorphicus* Corgosinho, Kihara, Schizas, Ostmann, Martínez Arbizu & Ivanenko, 2018 have morphological differences in terms of segmentations and setal armatures of P2–P4 (see Corgosinho et al. 2018: figs 9, 10). Because the copepodid stages of *Cerviniella* species have not been studied, it is unclear whether Becker (1974) analyzed immature specimens. The lack of accurate morphological information can lead to erroneous species identification; therefore, we excluded this species from the identification key provided above.

The female antennule of *Cerviniella* is typically five- or seven-segmented. The five-segmented antennule has an aesthetasc on the second segment, whereas the primitive antennule with seven segments has an aesthetasc on the third segment. The aesthetasc on the second segment is derived from the fusion of the second and third segments. Recently, Kihara and Martínez Arbizu (2012) reported a small aesthetasc present on the last segment in three species of *Cerviniella*, *C.arctica*, *C.danae*, and *C.hitoshii*. Two new Korean species also have this aesthetasc in the last antennular segment. In *C.lagarderei*, the last antennular segment has an aesthetasc-like element (see Bodin 1968: pl. VI). This aesthetasc was probably not detected in previous studies because of its slender and small appearance, which makes it different from the other setae. Overall, all species of *Cerviniella* have an additional aesthetasc, most likely located on the last segment of the antennule (see Bodin 1968: pl. VI).

Bodin (1968) described the female antennule of *C.langi* as seven-segmented and found that the last segment was broken (“le septième était brisé sur mon exemplaire”) (Bodin 1968: 10). However, the last segment is figured as possessing five well-developed and three small setae, similar to other *Cerviniella* species in his illustration (see Bodin 1968: pl. III). Assuming that his description is correct, only two lateral setae should be present on the last segment (i.e., the sixth segment) in his figure, considering the setal armature of other congeners. It implies that he probably examined the intact antennule of *C.langi*, not incomplete. Pending a reexamination of his type material, we here suggest that this deep-sea species has the six-segmented female antennule based on the figure (Bodin 1968: pl. III).

Apostolov (2011) provided an unclear description of the P3 and P4 setal armature of *C.abyssalis*. The armature complement of P3 exopod was described as “424” in the table with the armature formula of P1–P4 (Apostolov 2011: 96), but its armature was described as “425” in the comparison table of *Cerviniella* species (Apostolov 2011: 98); note that the species name *C.abyssalis* written erroneously as “*C.abyssalis*” in Apostolov’s (2011: 98) table. In his “Affinités” section, Apostolov (2011: 96) argued that *C.abyssalis* has the same armature as the P1 and P3 endopods (“1.121” and “1.220,” respectively) and the P1 and P4 exopods (“125” and “023,” respectively) in *C.bodini*. However, in Apostolov’s (2011: 98) table, the P4 exopod of *C.abyssalis* had a setal armature of “123.” Apostolov (2011) also mentioned that the setal armature of the P3 exopod of *C.abyssalis* includes 11 setae and spines, which differs from the ten setae and spines of *C.bodini*. However, in the accompanying figure of the P3 exopod, ten setae and spines are shown on the left, whereas 11 are displayed on the right. Additionally, the P4 of *C.abyssalis* is not illustrated, but Apostolov (2011) wrote that the P4 endopod was not observed. In contrast, the P4 endopod was marked as “présent?” in the armature formula of this species (see Apostolov 2011: 96), and a score of “1” was assigned for the corresponding ramus in the comparison table of the *Cerviniella* species (see Apostolov 2011: 98). Thus, the morphology of *C.abyssalis* remains unclear. Kihara and Martínez Arbizu (2012) provided the most recent comparison of the setal armature of *Cerviniella* species but did not incorporate the findings by Apostolov (2011).

Intraspecific variability is common in the thoracic legs of harpacticoids, as observed in multiple benthic species, such as *Argestesangolaensis* George, 2008, *Mesocletodeselmari* Menzel, 2011 (Argestidae), *Normanellaspinosa* Kim, Cho & Lee, 2014 (Normanellidae), and *Quinquelaophonteenormis* Kim, Nam & Lee, 2020 (Laophontidae) (George 2008; Menzel 2011; Kim et al. 2014; Kim et al. 2020). Intraspecific variability in the genus *Cerviniella* has been reported by Apostolov (2011) and Kihara and Martínez Arbizu (2012). As mentioned previously, Apostolov (2011) observed different numbers of setae on the left and right P3 exopods of a single specimen of *C.abyssalis*. Kihara and Martínez Arbizu (2012) identified an additional inner seta of the P3enp-2 in *C.arctica* (Kihara and Martínez Arbizu 2012: 16, fig. 10C) and *C.danae* (Kihara and Martínez Arbizu 2012: 10, fig. 6A). Additionally, the new species, *C.bisegmenta* sp. nov., showed intraspecific variability in the number of setae on P2enp-2, ranging from two to four (see “Variability” in *C.bisegmenta* sp. nov.). Furthermore, some specimens showed different numbers of setae on the left and right sides, suggesting the need for careful observation of the P1–P4 armature formula.

To establish the exact diagnosis of *Cerviniella*, it will be necessary to re-examine the ambiguous characteristics, including interspecific variability. There are 13 known species of *Cerviniella*, including four Arctic, six Atlantic, one Mediterranean, one Indian, and one from the Pacific Ocean. The discovery of two new species in the Korean Yellow Sea suggests that *Cerviniella* has a wider distribution than previously known. With the discovery of these new species, the family Aegisthidae and genus *Cerviniella* are reported in the Korean fauna here for the first time.

## Supplementary Material

XML Treatment for Cerviniella

XML Treatment for Cerviniella bisegmenta

XML Treatment for Cerviniella permixta

## ﻿Additional information

### Author ORCIDs

Kyuhee Cho https://orcid.org/0000-0002-5006-5713

Jong Guk Kim https://orcid.org/0000-0001-5299-9838

Jimin Lee https://orcid.org/0000-0001-9004-8275

### Data availability

All of the data that support the findings of this study are available in the main text.
